# Hematogones With Lambda Light Chain Restriction in a 4-Year-Old Boy With Burkitt Lymphoma: A Potential Diagnostic Pitfall

**DOI:** 10.1093/labmed/lmw009

**Published:** 2016-04-11

**Authors:** Tesha Guillory, Shiyong Li, Daniel J. Bergsagel, Elizabeth Weinzierl, Silvia T. Bunting

**Affiliations:** ^1^Department of Pathology, Emory University, Atlanta, GA,; ^2^Division of Hematology/Oncology, Department of Pediatrics, AFLAC Cancer and Blood Disorder Center, Emory University, Atlanta, GA,; ^3^Department of Pathology, Children’s Healthcare of Atlanta, Emory University School of Medicine, Atlanta, GA

**Keywords:** flow cytometry, hematopathology, hematogones, light-chain restriction, Lambda light chain, lymphoproliferative disorders

## Abstract

Hematogones are immature normal B cell precursors with a characteristic immunophenotype profile on flow cytometry that typically do not express surface immunoglobulin light chains. In this report, we describe a case in which the hematogones exhibit light chain restriction. Our patient was a 4-year-old boy with a complicated medical history involving treatment for a presumed bilateral Wilms tumor of the kidney that on later resection was diagnosed as Burkitt lymphoma. Flow cytometry analysis of his bone marrow revealed a small distinct population of cells expressing dim cluster of differentiation (CD)10, CD19, CD22, CD38, dim CD58, human leukocyte antigen–D related (HLA-DR), and dim CD45, which are characteristic of hematogones. These cells, however, demonstrated dim surface immunoglobulin lambda light-chain restriction. Molecular study results for immunoglobulin heavy and kappa light-chain gene rearrangements were negative. We present this case to raise awareness of the potential pitfalls of working up bone marrow for involvement by B cell lymphoproliferative disorder.

Hematogones are immature B-lymphocyte precursors that are found in the bone marrow and are most prominently observed in young children. As many as 80% of bone marrow specimens have been found to contain hematogones.^1^ Morphologically, hematogones are small uniform cells with round nuclear contour, condensed/smudgy chromatin, and scant cytoplasm. They do not have visible nucleoli and are typically located in the marrow interstitium or dispersed throughout the marrow. Hematogones can be identified by flow cytometry based on their light scatter characteristics and immunophenotype. At the most immature stage (stage I), hematogones have low side scatter and dim cluster of differentiation (CD)45 expression and commonly test positive for CD19, CD10, CD34, CD38, CD58, and terminal deoxynucleotidyl transferase (TdT). In their more mature stage (stage II), they start to acquire CD20 that ranges from dim to near the same intensity of the mature B lymphocytes; simultaneously, they lose CD34 and TdT. In most circumstances these cells do not express surface immunoglobulin light chains. In this report, we describe a case in which the hematogones showed dim lambda light chain restriction.

## Case Report

In July 2014, a 4-year-old boy arrived at the emergency department of Children’s Healthcare of Atlanta, Atlanta, GA with chronic urticaria (of 2 to 3 months duration) on the extensors and trunk, as well as worsening abdominal distention. He was also experiencing low-grade fevers and fatigue. The boy had no significant past medical or family history. Physical examination revealed hepatosplenomegaly. Laboratory studies revealed anemia and renal insufficiency. A magnetic resonance imaging (MRI) study of the abdomen showed a large mass in the upper pole of the right kidney that had extended through the renal capsule; these findings were radiologically interpreted as being consistent with Wilms tumor. There were multiple lobulated masses in the middle to inferior pole of the right kidney and throughout the left kidney, which we radiologically interpreted as being consistent with nephroblastomatosis. The patient was started on a course of emergent therapy with vincristine/actinomycin based on radiographic diagnosis and enrolled in study AREN0534 (radiographic diagnosis only; no biopsy per study). He developed significant tumor lysis. Children’s Oncology Group Quality Assurance Review Center (COG QARC) imaging review confirmed the radiologic interpretation of innumerable bilateral lesions compatible with diffuse rests with superimposed tumor. An ultrasound performed 2 weeks into the course of therapy demonstrated significant response (7.0 x 6.2 x 5.0 cm compared with 14 x 11 x 11 cm in a previous study). The patient was readmitted to the hospital for abdominal pain and dehydration with week 3 of vincristine. Due to the unusual complication, the patient underwent a partial right nephrectomy and biopsy of the left kidney after 6 weeks of therapy. Pathologic analysis of the right nephrectomy specimen demonstrated Burkitt lymphoma with no evidence of Wilms tumor. The left kidney showed mild interstitial fibrosis and interstitial nephritis; no tumor was observed. The patient was treated with Burkitt lymphoma therapy, which included rituximab, and he had a complete response. The patient remains free of disease at the publication of this report, 18 months since the diagnosis of Burkitt lymphoma.

## Histomorphologic Findings

**Image 1:**
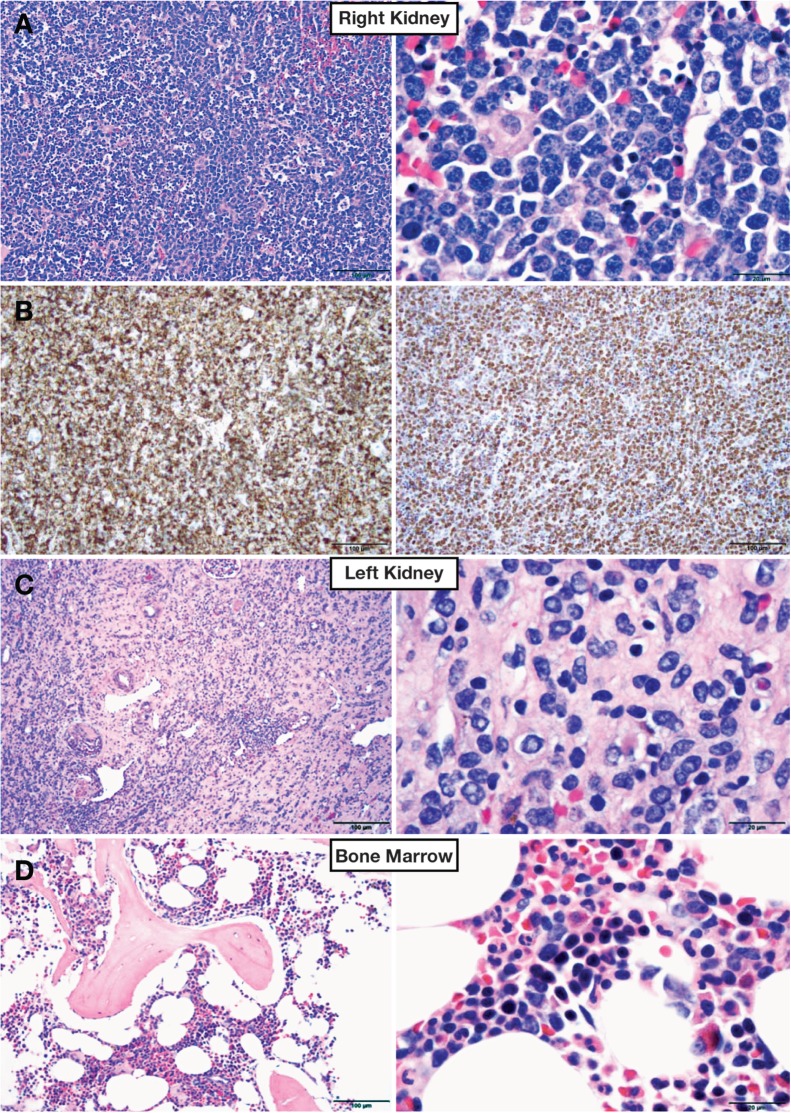
Histology of representative sections of the bilateral renal biopsy and bone-marrow biopsy from our patient, a 4-year-old boy. **A**, Hematoxylin-eosin (H&E) staining of the right kidney (original magnification left panel x20; right panel x1000). **B**, Immunohistochemical stains for CD20 (left panel) and Ki-67 (right panel) of the right kidney (original magnification x20 for both panels). **C**, Hematoxylin-eosin (H&E) staining of the left kidney (original magnification left panel x20; right panel x1000). **D**, Hematoxylin-eosin (H&E) staining of the bone-marrow core biopsy (original magnification left panel x20; right panel x1000).

Hematoxylin-eosin (H&E) sectioning of the right nephrectomy specimen demonstrated a relatively well-circumscribed proliferation of medium-sized homogeneous cells with stippled chromatin and scant cytoplasm. Numerous apoptotic bodies were present in a background of numerous histiocytes, which resulted in a starry-sky appearance (**Image 1A**) . The tumor cells tested diffusely positive for CD20 and CD10, and Ki-67 immunostaining highlighted more than 95% of tumor cells (**Image 1****B**). Bcl-2 tested positive in a small subset of the tumor, and a TdT stain yielded a negative result.

H&E sectioning of the left renal biopsy demonstrated a fragment of kidney with mild interstitial fibrosis, interstitial nephritis, and mild tubular atrophy. No nephrogenic rests were observed (**Image 1****C**).

Bone marrow biopsy revealed slightly hypocellular marrow with scattered small interstitial lymphoid cells. No evidence of Burkitt lymphoma was present (**Image 1****D**).

## Fluorescence In Situ Hybridization

**Image 2:**
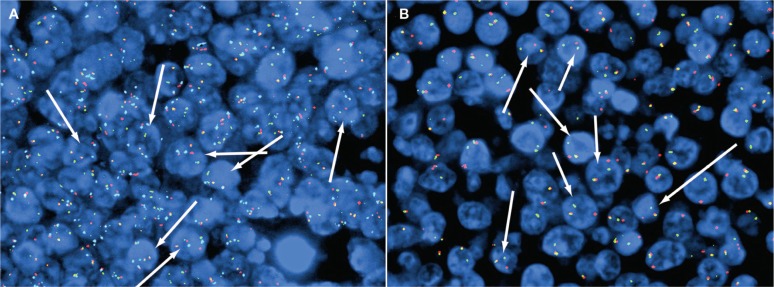
Imaging of fluorescence in-situ hybridization studies performed on the paraffin section of the right renal mass of our patient, a 4-year-old boy. **A**, t(8;14) translocation involving cellular MYC (c-MYC) and immunoglobulin (Ig)H. Arrows show the Burkitt lymphoma cells with 2 fusions, 1 orange, 1 green, and 2 aqua signals. **B**, Cellular MYC (c-MYC) break-apart study results. Arrows show the Burkitt lymphoma cells with 1 orange, 1 green, and 1 fusion signals.

We performed fluorescence in situ hybridization (FISH) testing on formalin-fixed, paraffin-embedded tissue from the right renal tumor of the patient. Two DNA probe sets (Abbott Laboratories, Inc) were used. For the cellular MYC (c-MYC) break-apart study, DNA probes flank the MYC locus with centromeric 5’ region in spectrum orange and the telomeric 3’ region in spectrum green. For the t(8; 14) fusion study, DNA probes are labeled with spectrum aqua for the centromeric region CEP8, spectrum orange for 8q24 MYC, and spectrum green for 14q32 IgH. Approximately 200 interphase cells were evaluated for each probe set. The test yielded positive results for a rearrangement of MYC in 71% of the cells and positive results for t(8q; 14q) in 70% of the cells. These findings are diagnostic for Burkitt lymphoma (**Image 2**). FISH assay for c-myc was not performed on the core biopsy due to decalcification.

## Flow Cytometric Immunophenotyping

Bone marrow aspirate from the patient was collected in ethylenediaminetetraacetic acid (EDTA) anticoagulant. The specimen was processed using a whole blood lysing system with ammonium chloride and stained with fluorochrome-conjugated antibodies. The list of antibodies used and the antibody combination in each tube are shown in Table 1. The data were collected using the Becton Dickinson Canto II instrument (Becton Dickinson and Company), equipped with 488 nm argon and 633 nm HeNe lasers. The analysis was performed using Data-Interpolating Variational Analysis (DIVA) BD FACSDiva software, version 6.0.

**Table 1:** Antibodies Used and Antibody Combination Per Tube, Used With Specimens From the Patient, a 4-Year-Old Boy

Tube	FITC	PE	Per-CP	APC
1	IgG1	IgG1	CD45	IgG1
2	CD14	CD13	CD45	CD34
3	HLA-DR	CD22	CD45	CD33
4	CD3	CD4	CD45	CD8
5	CD2	CD7	CD45	CD5
6	CD10	CD19	CD45	CD34
7	CD10	CD20	CD45	CD19
8	CD58	CD19	CD45	CD38
9	Kappa	Lambda	CD45	CD19

FITC, fluorescein isothiocyanate; PE, Phycoerythrin; Per-CP, peridinin chlorophyll protein; APC, Allophycocyanin; Ig, immunoglobulin; CD, cluster of differentiation; HLA-DR, human leukocyte antigen–D related.

## Immunoglobulin Heavy and Kappa Light Chain Gene Rearrangement

We collected the bone marrow aspirate from the patient in EDTA anticoagulant. DNA was extracted using EZ-1 DNA Investigator Kit (QIAGEN) extraction. The formalin-fixed paraffin-embedded (FFPE) tissue block from the kidney resection specimen was cut, and DNA was extracted with the QIAGEN DNA (QIAGEN) extraction procedure. Polymerase chain reaction (PCR) was performed using InVivoScribe PCR reagents (Invivoscribe) and AmpliTaq Gold DNA Polymerase (Thermo Fisher Scientific Inc). We performed capillary electrophoresis and fluorescence detection with the ABI Prism 3130 Genetic Analyzer System (Applied Biosystems, Inc), and the PCR products were analyzed using the ABI Prism GeneMapper Analysis Software, version 3.7 (Applied Biosystems, Inc).

## Discussion

**Figure 1:**
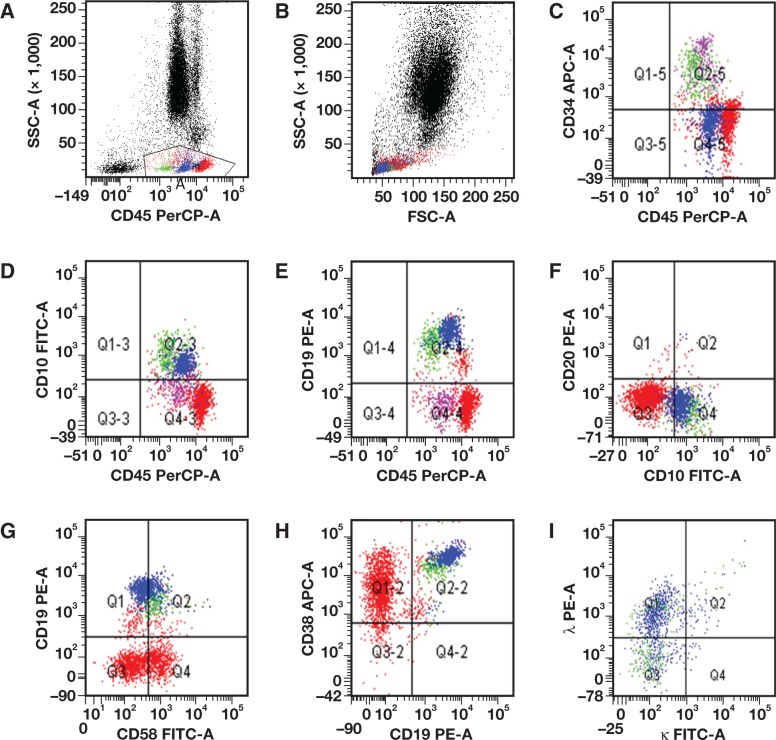
Representative scatter plots of flow cytometric immunophenotyping on the bone-marrow specimen from our patient, a 4-year-old boy. Hematogones, indicated by green (stage I) and blue (stage II) dots, demonstrated lambda light-chain restriction. Purple dots indicate normal myeloblasts, and red dots indicate T lymphocytes and few normal B cells. **A**, CD45 versus side scatter dot plot; the gate indicates the cells of interest. **B**, Forward scatter versus side scatter dot plot. **C**, CD34 versus CD45 dot plot; The stage I hematogones (green) are positive for CD34, while stage II (blue). The purple dots represent normal myeloblasts which has brighter CD34 than the stage I hematogones (green). **D**, CD10 versus CD45 dot plot; Both stage I (green) and stage II hematogones (blue) are positive for CD10. Stage I hematogones have brighter CD10 than stage II hematogones. **E**, CD19 versus CD45 dot plot; The normal B cells (red in the right upper quardrant) show slightly dimmer CD19 expression, which is seen the patient receiving rituximab therapy. **F**, CD20 versus CD10 dot plot. The stage II hematogones did not demonstrate the typical gradually increasing expression of CD20 due to rituximab therapy. **G**, CD19 versus CD58 dot plot; stage I and stage II hematogones show the typical pattern of C19 and CD58 expression, ie, stage II hematogones show slightly decreased CD58 expression than the stage I hematogones, while stage II hematogones have slightly increased CD19 expression. **H**, CD38 versus CD19 dot plot; the stage I and stage II hematogones show the typical pattern. **I**, Surface kappa versus lambda light chain dot plot. A subpopulation of both stage I and stage II hematogones show lambda light chain restriction, while a subpopulation of the stage I and stage II hematogones show lack of either kappa or lambda light chains.

**Figure 2:**
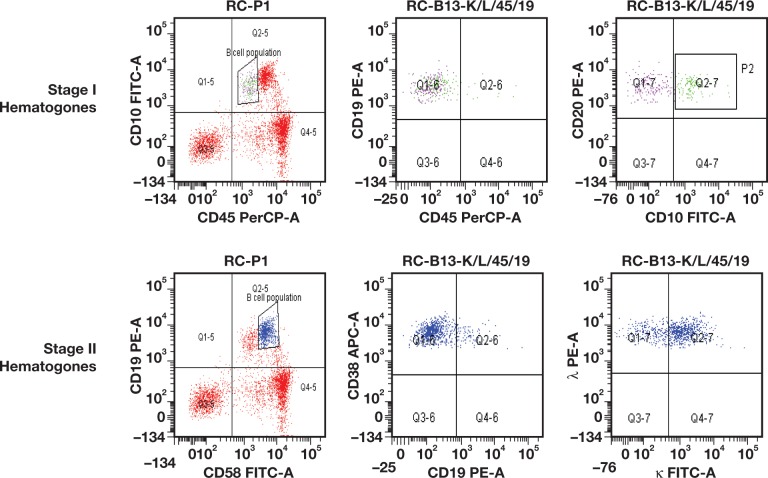
Additional dot plots of flow cytometric immunophenotype of the stage I (green) and stage II (blue) population, from the bone marrow specimen from our patient, a 4-year-old boy.

**Figure 3:**
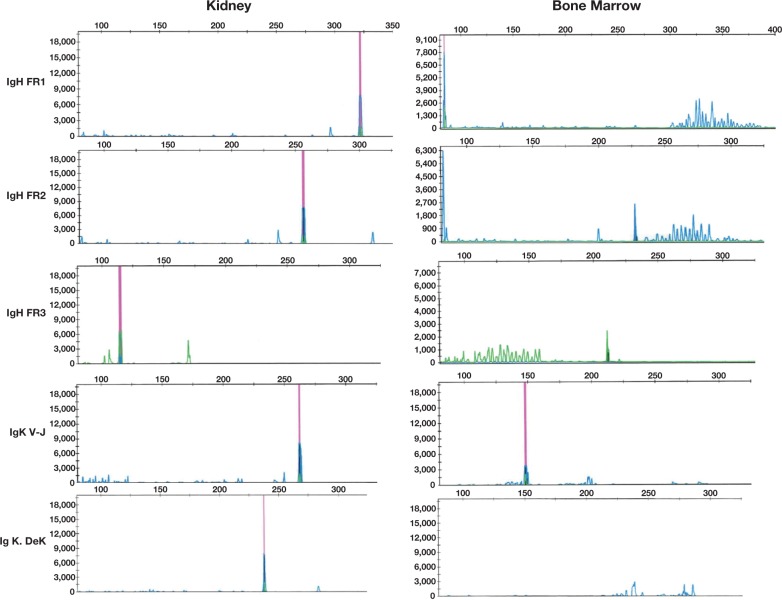
Electropherogram of immunoglobulin and kappa light chain gene rearrangement by polymerase chain reaction (PCR). Clonal rearrangements of immunoglobulin (Ig) heavy and kappa light chain genes were present in the kidney specimen (a distinct peak in all regions analyzed, left panel) but absent in the bone marrow specimen (normal polyclonal background peaks in all regions analyzed, right panel) from our patient, a 4-year-old boy.

Staging of the bone marrow biopsy material revealed normal trilineage hematopoiesis with erythroid hyperplasia. Flow cytometry analysis demonstrated a distinct population (4%) of cells with low-forward and right-angle scatters. These cells expressed dim CD10, CD19, CD22, CD38, dim CD58, HLA-DR, and dim CD45; the cells tested negative for CD20 due to rituximab ingested the day before the biopsy. This population also exhibited lambda immunoglobulin light chain restriction (Figure 1 and Figure 2). These cells were classified as hematogones with lambda light chain restriction. Subsequently, we performed immunoglobulin heavy and kappa light chain gene rearrangements by PCR on the bone marrow aspirate and showed there was no clonal population identified (Figure 3, left panel). Concurrent molecular testing performed on the right kidney showed distinct peaks consistent with clonal rearrangements of immunoglobulin and kappa light chain genes (Figure 3, right panel). There was no bon marrow specimen submitted for karyotype or florescence in situ hybridization study. We could not perform the c-MYC translocation study on the decalcified core biopsy. However, we believe that the immunoglobulin heavy chain and kappa light chain gene rearrangement studies by PCR are the more sensitive test for detecting this specific molecular abnormality. Our findings showed no bone marrow involvement by Burkitt lymphoma.

Hematogones are immature B-cell precursors found in the bone marrow. They are more commonly detected in higher numbers in neonates and umbilical cord blood specimens, and their numbers typically decrease with age. However, they can also be observed in patients recovering from bone marrow transplantation or chemotherapy. An increased number of hematogones can be seen in a variety of benign and malignant conditions,^2^ including autoimmune conditions, idiopathic thrombocytopenic purpura (ITP), and certain viral infections. Hematogones are markedly decreased in patients with myelodysplastic syndrome and severe aplastic anemia.^3^ Hematogones can also be detected in healthy adults, but typically, these occur in much smaller quantities. Currently, there are no guidelines for an appropriate amount of hematogones in the bone marrow.

Hematogones are typically categorized into 3 stages, based on maturity and immunophenotype. Stage I hematogones are the most immature and express TdT, CD34, CD10, CD38, and dim CD19. Stage II hematogones show a loss of TdT and CD34, dim CD45, dimmer CD10, and CD38, and they gradually acquire increasing intensity of CD20 and show more CD45 expression compared with the stage I hematogones. The most mature hematogones, namely, those in stage III, have the same intensity of the mature B cells and lose CD10 expression. In typical flow cytometry analysis, the stage 3 hematogones are merged with the mature B lymphocytes. The significance of identifying hematogones in the bone marrow is to distinguish them from lymphoblasts, especially for minimal residual disease detection. Via flow cytometry light scatter plotting, hematogones often have a distinctive 3-cluster pattern that correlates to the 3 stages of maturity, with a characteristic immunophenotypic pattern demonstrating the normal maturation sequence. This is a helpful distinguishing feature, although it is not always present.^1^ B-cell acute lymphocytic leukemia (B-ALL) lymphoblasts usually have an aberrant immunophenotype that is inconsistent with the normal B cell maturation sequence; they can also acquire aberrant markers such as myeloid or T cell markers.

Another characteristic feature of hematogones is their lack of surface immunoglobulin. However, both our case study and several others have shown that in rare circumstances, hematogones can demonstrate surface light chain restriction, which could present a diagnostic challenge in a staging-based bone marrow study for non-Hodgkin lymphoma. In a large study published as an abstract by Challagundla et al,^4^ in the staging of bone marrow from patients with B-cell neoplasms,^4^ hematogones showed a marked light chain bias. Of the 11 cases in which hematogones were identified, 8 cases were kappa restricted and 3 cases were lambda restricted. Eight of the patients did not have bone marrow involvement via lymphoma. Seven of those 8 cases had flow cytometric immunophenotyping of the lymphomas and the hematogones. Five of the 7 cases showed the same light chain restriction, whereas 2 of the 7 cases showed different light chain restriction in the lymphomas and in the hematogones. This finding shows the importance of properly identifying the presence of hematogones to avoid overdiagnosis of bone marrow involvement by lymphoma. An abstract by Setiadi et al^5^ reported 2 cases with light chain restriction in hematogones. The hematogones in these cases demonstrated lambda light-chain restriction.^5^ It should be emphasized that B-ALL lymphoblasts can also express surface light chains, particularly in cases with mixed-lineage leukemia (MLL) gene abnormalities.^6^^,^^7^

These findings, in addition to those we detail in the case described herein, demonstrate that in rare cases, hematogones can exhibit surface immunoglobulin expression. The exact mechanism for this expression and the clinical significance behind this finding are unknown. We speculate that this phenomenon is similar to those reported in pediatric patients with extranodal marginal zone hyperplasia in which there is immunophenotypic evidence of surface immunoglobulin lambda light chain restriction, whereas there is no molecular evidence of a clonal population.^8^ It has been hypothesized colloquially that there might be functional differences between the kappa- or lambda-expressing B cells. However, this finding could present a diagnostic pitfall, especially in staging bone marrow biopsies for non-Hodgkin lymphoma. 

## Abbreviations

CD: cluster of differentiation
Tdt: terminal deoxynucleotidyl transferase
MRI: magnetic resonance imaging
COG QARC: Children’s Oncology Group Quality Assurance Review Center
H&E: hematoxylin-eosin
FISH: fluorescence in situ hybridization
c-MYC: cellular MYC
FFPE: formalin-fixed paraffin-embedded
PCR: polymerase chain reaction
ITP: idiopathic thrombocytopenic purpura
B-ALL: B-cell acute lymphocytic leukemia
MLL: mixed-lineage leukemia gene
FITC: fluorescein isothiocyanate
PE: Phycoerythrin
Per-CP: peridinin chlorophyll protein
APC: Allophycocyanin
Ig: immunoglobulin
